# Estimating age-dependent per-encounter chlamydia trachomatis acquisition risk via a Markov-based state-transition model

**DOI:** 10.1186/2043-9113-4-7

**Published:** 2014-04-25

**Authors:** Yu Teng, Nan Kong, Wanzhu Tu

**Affiliations:** 1 Weldon School of Biomedical Engineering, Purdue University, 206 S. Martin Jischke Dr, West Lafayette, IN 47907, USA; 2 Division of Biostatistics, School of Medicine, Indiana University, 340 W 10th Street, Indianapolis, IN 46202, USA

**Keywords:** Chlamydial infection, Acquisition risk, Transmission probability, Parameter estimation, State transition model

## Abstract

**Background:**

Chlamydial infection is a common bacterial sexually transmitted infection worldwide, caused by *C. trachomatis*. The screening for *C. trachomatis* has been proven to be successful. However, such success is not fully realized through tailoring the recommended screening strategies for different age groups. This is partly due to the knowledge gap in understanding how the infection is correlated with age. In this paper, we estimate age-dependent risks of acquiring *C. trachomatis* by adolescent women via unprotected heterosexual acts.

**Methods:**

We develop a time-varying Markov state-transition model and compute the incidences of chlamydial infection at discrete age points by simulating the state-transition model with candidate per-encounter acquisition risks and sampled numbers of unit-time unprotected coital events at different age points. We solve an optimization problem to identify the age-dependent estimates that offer the closest matches to the observed infection incidences. We also investigate the impact of antimicrobial treatment effectiveness on the parameter estimates and the differences between the acquisition risks for the first-time infections and repeated infections.

**Results:**

Our case study supports the beliefs that age is an inverse predictor of *C. trachomatis* transmission and that protective immunity developed after initial infection is only partial.

**Conclusions:**

Our modeling method offers a flexible and expandable platform for investigating STI transmission.

## Background

Chlamydial infection, caused by the bacterium*, C. trachomatis*, is a commonly reported sexually transmitted infection (STI) worldwide [1]. It can be accurately diagnosed and effectively cured if being treated promptly [2-4]. On the other hand, the infection may go unnoticed for many years and consequently lead to severe morbidities, including pelvic inflammatory disease, ectopic pregnancy, tubal pregnancy, preterm birth, and increased susceptibility of HIV infection [5-13]. Therefore, it is important to the high-risk individuals and social groups as well as societies in general to schedule screening tests at the right time.

The above clinical facts promote the model-based analysis of screening programs for chlamydial infections [14-19]. Based on the evidence of high *C. trachomatis* prevalence among adolescents and remaining risk for repeated infection among those who were recently treated for infection [20-27], routine population screening for female adolescents, especially those who were recently infected, has been suggested to be cost-effective, and in some cases, cost-saving [18,28-31]. At present, some routine screening strategies are endorsed by clinical practice guidelines [32-34] and recommended to adolescents during their health visits [5,35,36]. However, when tailoring such strategies (e.g., specifying the screening frequency) with respect to age and prior infection status, we face the challenge of lacking reliable epidemiological data. Understanding the acquisition risk differences with respect to age and prior infection status may offer insights into the mechanism of *C. trachomatis* acquisition and chlamydial infection. Such understanding will lead to more detailed model-based economic studies on the effectiveness and cost-effectiveness of screening strategies, which has the potential to further improve the prevention of chlamydial infection.

In this paper, we use observational infection data to estimate age-dependent per-encounter *C. trachomatis* acquisition risks, i.e., the probability that a female subject is infected with *C. trachomatis* through an unprotected coital event. For brevity, we use acquisition risk for referring to per-encounter *C. trachomatis* acquisition risk. It is unethical to design controlled experiments that expose human subjects to infectious pathogens. This challenge is alleviated with observational studies and model-based studies. Katz [37] and Tu et al. [38] used cross-sectional data and longitudinal data to estimate the acquisition risk, respectively. The estimation was also described in several model-based studies for screening program/strategy evaluation. Kretzschmar et al. [14] developed an individual-based stochastic simulation model to describe the spread of *C. trachomatis* in a heterosexual population with a highly sexually active core group. The authors used Monte-Carlo simulation to estimate the daily transmission rates. The data source used in this paper for sexual behavior and partnerships were based on a survey conducted in the Netherlands in 1989 [39]. Kretzschmar et al. [15] used the simulation model in [14], acquired the per partnership transmission probability from [40], and applied the method in [37] to estimate the per-act transmission probability. Turner [17] used a more comprehensive stochastic network model based on Ghani et al. [41] for the estimation. The network includes not only disease transmission and recovery but also dynamic partnership choice, formation, and dissolution. The transmission probabilities per sex act were estimated by systematic fitting to a variety of appropriate UK-based data sources [42,43]. Other studies on the transmissibility of *C. trachomatis* include Gray et al. [44], Lycke et al. [45], Ruijs et al. [46], and Vickerman et al. [47]. However, none of the papers above investigated the age- and prior-infection-dependency on *C. trachomatis* transmissibility. Additionally, many model-based studies used multiple data sources and relied on expert opinion driven model assumptions. For a list of works on estimating or using *C. trachomatis* transmission probabilities, we refer to Additional file 1.

To estimate age-dependent acquisition risks, we developed a Markov-based individual state-transition model that describes the changes in states of infection for each subject over time. The transition probabilities in the model are time-varying. In addition, we extended our model to investigate the difference on the acquisition risk that leads to a first-time infection and leads to a repeated infection. Furthermore, we varied the effectiveness of antimicrobial treatment to assess its impact on the acquisition risk estimates. Methodologically, the approach proposed is a novel application of time-varying Markov modeling with longitudinally observed infection data. It allows us to assess the probability of failures in antimicrobial treatment. To our knowledge, only Tu et al. [38] applied a similar approach of using longitudinal data to estimate the transmissibility of *C. trachomatis*. However, they did not consider the potential age-dependency in the changes on the infection state nor differentiate first-time infection and repeated infection. While there are many articles estimating the transmissibility of HIV/AIDS (e.g. [48,49]), few statistical approaches (e.g. [38]) were developed for bacterial STI with additional data and methodological challenges. Unlike HIV/AIDS, infections with STI bacteria are routinely treated and effectively cured. This causes a shift in the state of infection and poses greater methodological challenges on quantifying the transmissibility. Meanwhile, frequently measured infection data are needed to capture such infection dynamics. To our knowledge, few studies have been designed to collect such data. In the next section, we describe an observational study providing ideal data for our research.

The acquisition risk is a population-specific quantity, which reflects not only the transmissibility of the *C. trachomatis* organism but also the organism’s prevalence in the male partner population. Clearly, higher acquisition risks are associated with the organism being more transmissible and being more prevalent among male partners. Given *C. trachomatis* prevalence in the male partner population, one can quantify the male-to-female *C. trachomatis* transmission probability for young women within a particular age group to be the ratio between the per-encounter *C. trachomatis* acquisition risk within the particular age group and the prevalence among the male partners that are associated with the young women of that age group, i.e., per-encounter *C. trachomatis* acquisition risk = transmission probability × prevalence in the male partner population. For our case study, we used the observational data on recurrent sexually transmitted disease among the recruited adolescent women. Because the exact prevalence of *C. trachomatis* infection in the male partner population was not attained in the study used, we in this paper focus on the estimation of acquisition risks, which reflects the transmission risk presented in the male partner population.

The remainder of the paper is organized as follows. In Section 2, we describe the observational data set used in the parameter estimation. In Section 3, we describe our estimation method. In Section 4, we report our estimates based on the data set and discuss the results. We draw conclusions, discuss limitations, and outline future research in Section 5.

### Description of the analyzed data set

**Figure 1:**
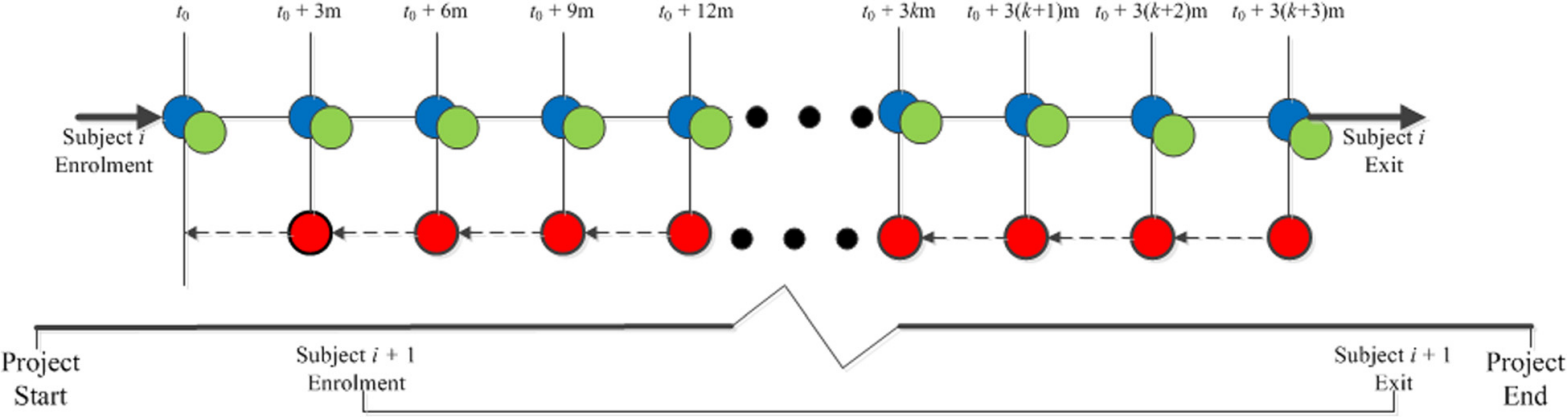
**Data collection scheme for the YWP:the blue dots indicate the determination of the STI status at enrollment and all subsequent quarterly visits;the light green dots indicate antimicrobial treatment of infected subjects at the visits;and the red dots indicate face-to-face interviews in which subjects reveal the number of unprotected coital events.**

The observational data used in our case study were collected through the “Young Women’s Project” (YWP), which is an epidemiological study of recurrent STI in adolescent women recruited from an inner city population that was at increased risk of STI. The YWP started its enrollment in 1999. Its study design and data collection provide a platform for the estimation of the *C. trachomatis* transmissibility. We present the data collection scheme in Figure 1.

In a nutshell, young women between the ages of 14 and 17 years old were identified and recruited by the project. The recruitment criteria used included 1) understanding English; 2) no serious mental or psychiatric health problems; and 3) visiting one of three participating primary care clinics. For those young women who met all the recruitment criteria, they would be approached by research staff and asked for enrollment. Note that these criteria do not depend on subjects’ prior sexual experience, which ensures the randomness of the cohort. To enroll an YWP study subject, she received initial interviews and underwent a pelvic examination, during which a cervical swab for STI testing was collected and analyzed with nucleic acid amplification test for *C. trachomatis*. Infected participants were treated while at the clinics or shortly after the test results became available. Enrolled participants were then asked to visit their clinics on a quarterly basis. At each follow-up visit during the study period, enrolled participants underwent STI testing and treatment. Also at each follow-up visit, the participants received follow-up interviews, in which they were queried about the number of unprotected coital events since previous visit. Most of the participations did not visit the clinic every quarter and left the project before its completion.

For our analysis, we collected 1173 quarterly test results from the first 200 participants who were never infected and have completed at least two follow-up visits. Their average enrollment age was 15 years with standard deviation of 1.1 years. These participants underwent averagely 5.86 visits, ranging from 2 to 18. They stayed in the project for 8.2 years maximally and 3.2 years on average. They reported averagely 14.3 quarterly unprotected coital events. *C. trachomatis* was detected from 208 of the quarterly swab samples, equivalent to 17%. For more information on the YWP data collection and the observational data set analyzed for this paper, we refer to Tu et al. [38].

### Description of the estimation method

**Figure 2:**
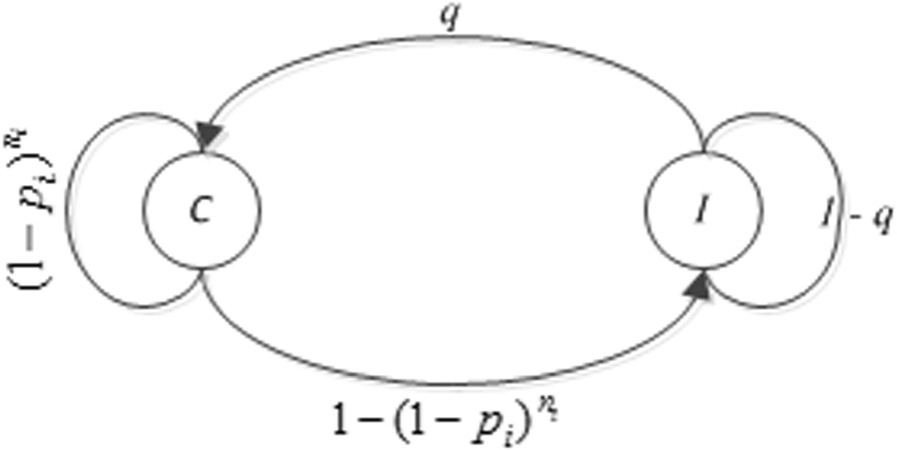
**State transition diagram of the age-dependent Markov model that captures the disease condition dynamics for chlamydial infection and curing at discrete age point** ***i*** **= 0, 1, …,** ***N*– 1.**

For the estimation, we first developed an age-dependent Markov state-transition model that depicts the disease condition dynamics for each female individual (Figure 2). In the model, we let *C* and *I* be the states where a female subject does not and does have *C. trachomatis*, respectively. Without loss of generality, we assume that the age range we study is [*T*_1_, *T*_2_]. We also assume Δ*t* to be the unit-time interval during which no state transition occurs and all transition rates remain the same. With the YWP, the smallest length for Δ*t* is set to be a quarter year, the time interval between two consecutive visits. Given the studied age range [*T*_1_, *T*_2_] and the unit-time interval Δ*t*, we index the discrete age points to be i = 0, 1, ⌈(*T*_2_ ‒ *T*_1_)/Δ*t*⌉ with 0 indexing *T*_1_ and *N* ≡ ⌈(*T*_2_ − *T*_1_)/Δ*t*⌉ indexing *T*_2_. We denote *p*_*i*_ to be a constant acquisition risk between discrete age points *i* and *i* + 1 for *i* = 0, 1, …, *N* - 1. That is, when a subject of age point *i* is at state *C*, we assume that the subject follows a constant probability to transition to state *I* with unprotected coital events between the two consecutive age points. Once a subject is at state *I* at age point *i*, the only reason she does not transition back to state *C* at the next discrete time point is due to the ineffectiveness of the antimicrobial treatment. We use *q* to measure the treatment effectiveness with the assumption that this quantity is constant irrespective of the age. That is, *q* is the probability that a subject transitions to state *C* given she is currently at state *I*. Lastly, we denote *t*_en_ ≥ *T*_1_ and *t*_ex_ ≤ *T*_2_ to be the entry and exit ages of a female subject, respectively. We can conveniently map the two age values to two age point indices between 0 to *N*. We call them *i*_*a*_ ≥ 0 and *i*_*b*_ ≤ *N*.

Let *s*_*i*_ be the state of a female subject between age point *i* and *i* + 1 with *i*_*a*_ ≤ *i* <*i*_*b*_. Let *n*_*i*_ be the number of unprotected coital events with an infected partner during the same period. Assuming that the probability that a female subject acquires *C. trachomatis* is independent between any two unprotected coital events, the transition probabilities are presented as: Pr (*s*_*i*+1_ = *C* | *s*_*i*_ = *C*) = ${\left(1-p_{i}\right)}^{n_{i}}$, which means that to ensure no infection, the subject does not acquire *C. trachomatis* in any of the unprotected coital events during the period. Here we also assume a female subject has zero probability of acquiring *C. trachomatis* from a protected coital event. Note that a similar idea can be found in Katz [37]. It is easy to determine the other transition probabilities in the state-transition model.

We next developed a cubic spline model, based on the longitudinal study in Tu et al. [38], to predict the number of unprotected coital events with an infected partner during the next quarter year at age *t* (in years). The model is presented as:

$$n\left(t\right)=\exp\;\left\{\beta_{0}+\beta_{1}t+\beta_{2}t^{2}+\beta_{3}t^{3}+\sum_{i=1}^{N}u_{i}{\left({\left(t-k_{i}\right)}_{+}\right)}^{3}\right\}.$$

A cubic spline model is a polynomial function that is piecewise-defined and possesses smoothness of order 3. For each discrete age point *i* = 0, 1,.., *N*-1, we can use its corresponding age, denoted by *t*_*i*_, to calculate *n*(*t*_*i*_) and specify *n*_*i*_. The expression (*a*)_+_ indicates the value of *a* is kept when it is nonnegative and its value is set to be 0 when negative. Given the data availability, the model shows good fit for the age range between years of 15 and 24.75. In the model, *N* indicates the number of knots and is set to be 40. The parameters *k*_*i*_, termed knots, indicate age points between 14.5 and 24.5. These knots are the places in the model where the polynomial pieces connect. The parameters *u*_*i*_, together with the base function *β*_0_ + *β*_1_*t* + *β*_2_*t*^2^ + *β*_3_*t*^3^, ensure that the estimates of the spline model match the corresponding observations exactly at those age points. We report the model parameter values in Additional file 2.

As an extension to incorporate cohort variation on the number of unprotected coital events, we assume the intercept (i.e., the first term *β*_0_) of the cubic spline model for each simulated subject to be normally distributed with mean being the intercept from the original model and standard deviation being a percentage of the mean. Once the intercept was sampled for each simulated subject, we adjusted the corresponding cubic spline model but kept the values of the knots the same for all the subjects to ensure the necessary correlation among different time points. Thus the extended stochastic model is presented as:

$$n\left(t\right)=\exp\;\left\{u_{0}+\beta_{0}+\beta_{1}t+\beta_{2}t^{2}+\beta_{3}t^{3}+\sum_{i=1}^{N}u_{i}{\left({\left(t-k_{i}\right)}_{+}\right)}^{3}\right\},$$

where *u*_0_ follows a normal distribution as *u*_0_ ~ *N*(0, (*ρ × β*_0_)^2^) with 0 <*ρ* ≤ 1.

To estimate age-dependent acquisition risks, we applied a reverse engineering approach with real age-specific chlamydial infection incidences extracted from the observational data. For any candidate age-dependent acquisition risk profile, we simulated the infection incidence given a collection of hypothetical female subjects, each of which is assigned a random enrollment age and a random exit age, based on the same observational data. For each subject, we also followed the stochastic model above to uniquely determine for each hypothetical subject the number of unprotected coital events in all discrete age points from the simulated enrollment to the simulated exit. With the simulation of each subject, we recorded the age points within which each infection occurred to the subject. Finally, we tallied the number of chlamydial infections for the entire simulated cohort within each age group.

We constructed an optimization problem to compute the acquisition risks with which the simulated incidences match the observed ones most closely. To present the optimization problem, let us introduce additional mathematical notation. We use $\bar{I_{i}}$ to denote the observed percentage of infected in the studied cohort for age group *i* = 1,…, *N*. We use *K* to denote the set that contains the indices of the simulation runs. For each age group *i*, we collect all hypothetical subjects that experienced *i* during their enrollment (i.e., the age corresponding to *i* is between the subject’s entry and exit ages). We denote *K*(*i*) ⊆ *K* to be the subset that contains the simulation run indices for such subjects. For each simulation run *k* ∈ *K*(*i*), we use *I*_*i*_(*k*) ∈ {0, 1} to indicate whether an infection occurs for age group *i* = 1,…,*N*. The objective of the optimization problem is to minimize the difference between $\frac{1}{\left|K\left(i\right)\right|}\sum_{k\in K\left(i\right)}I_{i}\left(k\right)$, the simulated percentage of infected, and $\bar{I_{i}}$ for each *i* = 1,…,*N*. The optimal solution specifies the age-dependent acquisition risk, which is denoted by $\bar{p}=\left({\bar{p}}_{1},\ldots ,{\bar{p}}_{N}\right)$.

**Figure 3:**
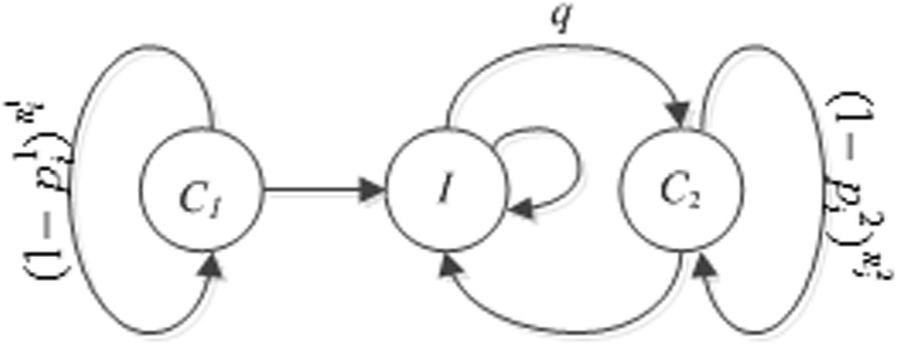
**State transition diagram of the time-varying Markov model that captures the disease dynamics with first-time chlamydial infection, curing, and repeated infection.** Note that the risks of chlamydial infection for the first time and repeated infection are different; and the sexual behaviors before and after the first-time infection are different as well.

We next considered the case where we distinguish the acquisition risks for first-time infection and repeated infection. This distinction is supported by the existing literature. Many studies, mostly involving women, have evaluated the risk of repeated infections during a period of observation and found that repeated infections are common during the first half a year after initially treated infections [21,22]. Some of these studies [25,26] also observed higher risk of repeated infections among younger women. We therefore extended the above Markov state-transition model (Figure 3). We let *C*_1_*and C*_2_ be the states where a female subject is cured from chlamydial infection for the first time and reinfection, respectively, due to unprotected coital events. Similar to the first model, we assumed that both risks are independent between any two unprotected coital events. We denote $p_{t}^{1}$ and $p_{t}^{2}$ to be the two corresponding acquisition risks, and denote $n_{t}^{1}$ and $n_{t}^{2}$ to be the numbers of unprotected coital events before the first infection and after, respectively. Then we have Pr (*s*_*i*+1_ = *I* | *s*_*i*_ = *C*_1_) = $1-{\left(1-p_{t}^{1}\right)}^{n_{t}^{1}}$ and Pr (*s*_*i+*1_ = *I* | *s*_*i*_ = *C*_2_) = $1-{\left(1-p_{t}^{1}\right)}^{n_{t}^{1}}$$1-{\left(1-p_{t}^{2}\right)}^{n_{t}^{2}}$. Once a subject has been infected, she will only transition between *C*_2_ and *I*, and never transition back to the state *C*_1_*.*

To estimate age-dependent acquisition risks, we again applied a reverse engineering approach. We constructed two optimization problems to compute the acquisition risks that lead to first-time infection and reinfection. With the acquisition risks, the simulated incidences (i.e., first-time infection incidence and reinfection incidence) should match the observed ones most closely. To present the optimization problems, let us introduce additional mathematical notation. We use ${\bar{S}}_{i}^{f}$ and ${\bar{S}}_{j}^{r}$, respectively, to denote the observed percentages of first-time infection and repeated infection in the studied cohort for age group *i*, *j* = 1,…, *N*. Similar to the above definition of *K*(*i*), we introduce *K*^*f*^(*i*) and *K*^*r*^(*i*). For each simulation run *k*, we use $S_{i}^{f}\left(k\right)$ and $S_{j}^{r}\left(k\right)$, respectively, to indicate whether a first-time infection and a repeated infection occurs within each age group. The objectives of the two optimization problems are to minimize $\frac{1}{\left|K^{f}\left(i\right)\right|}\sum_{k\in K^{f}\left(i\right)}S_{i}^{f}\left(k\right)-{\bar{S}}_{i}^{f}$ and $\frac{1}{\left|K^{r}\left(i\right)\right|}\sum_{k\in K^{r}\left(i\right)}S_{i}^{r}\left(i\right)-{\bar{S}}_{i}^{r}$ for each *i* = 1,…,*N*^*f*^ and *j* = 1,…, *N*^*r*^, respectively. The optimal solutions specify the age-dependent risks of acquiring *C. trachomatis* for the first time and reacquiring the bacterium, which are denoted by ${\bar{p}}^{f}=\left({\bar{p}}_{1}^{f},\ldots ,{\bar{p}}_{N^{f}}^{f}\right)$ and ${\bar{p}}^{r}=\left({\bar{p}}_{1}^{r},\ldots ,{\bar{p}}_{N^{r}}^{r}\right)$, respectively.

## Results

In our numerical study, we used observational data collected through the YWP on age-specific chlamydial infection rate for the first time since the previous visit, denoted by CT rate (i), and age-specific cumulative CT infection rate (including both first-time infection and repeated infection), denoted by *cum*_*CT*_*rate*(*i*), for each age point *i*. We also tallied the total number of visits made by subjects at each age group, denoted by *total*_*popu*(*i*). For the first Markov model, we have ${\bar{I}}_{i}$ = *CT*_*rate*(*i*) × *total*_*popu*(*i*). We considered the age range from 15 to 24.75 with 3 months as the length of the fixed unit-time interval. That is, *N* = 40. For the second Markov model, we have ${\bar{S}}_{i}^{f}$ = (1 – *cum*_*CT*_*rate*(*i*)) × *total*_*popu*(*i*) and ${\bar{S}}_{i}^{r}$ = (*cum*_*CT*_*rate*(*i*) – *CT*_*rate*(*i*)) × *total*_*popu*(*i*). Due to data scarcity on *cum*_*CT*_*rate*(*i*), we considered the age range from 17 to 22 with 6 months as the length of the fixed unit-time interval for the second model. That is, *N* = 10. For both Markov models, we used the cubic spline model described earlier to predict the number of unprotected coital events within each 3-month interval between age 15 and 25. We introduced randomness to the cubic spline model by assuming a normal distribution on the intercept (i.e., the first term *β*_0_) of the model and setting its standard deviation to be 10% of the intercept based on expert opinion, i.e., *ρ* = 10%. Then we drew a sample from the normal distribution in each simulation run. In both models, we also varied *q*, the antimicrobial treatment failure probability, to be 0%, 5%, or 10%. It is worth noting that the treatment failure probability may have significant variation among individual patients and clinics. That motivated us to conduct a sensitivity analysis. Lau and Qureshi [50] reported in a meta-analysis of randomized clinical trials that the failure probabilities of using azithromycin and doxycycline, the antibiotic considered in the YWP, to treat genital chlamydial infections were on average 3% and 2%, respectively. The treatment failure probability in a real-world setting is less defined and difficult to measure. A recent article (Tu et al. [38]) suggested using 10% as the expected chance of a treatment failure. These numbers helped us specify the probability range for our sensitivity analysis.

We ran the simulation 10,000 times with generation of 10,000 hypothetical subjects for the study of each case described above. We report in Tables 1 and 2 the means at selected age points. From Table 1, we observed that 1) $\bar{p}$ monotonically decreases as age increases; 2) $\bar{p}$ decreases as *q* increases. The first observation supports the evidence that the rate and prevalence of chlamydial infection are shown inversely related with age [51,52]. The second observation matches the intuition behind the relationship between treatment effectiveness and infection risk. If the treatment is less effective, the infected individual stays longer in state *I* and thus takes a longer period of time to be infected again. This leads to a lower acquisition risk to match the observational data. From Table 2, we observed 1) ${\bar{p}}^{r}$ is positive; 2) both ${\bar{p}}^{f}$ and ${\bar{p}}^{r}$ monotonically decrease as age increases, and 3) ${\bar{p}}^{r}$ decreases as *q* increases. The first observation support the concept that some degree of protective immunity against reinfection develops after first-time infection, although it appears to be partial at best [53,54]. The second observation supports the concept that acquired protective immunity may restrict chlamydia replication in older persons [55]. In other words, in addition to likely inverse relationship between age and unprotected sexual activity, organism load has been shown inversely related with age as well. As a result, many naive persons, considering themselves treated for initial infections successfully, may resume sexual activity at a level as active as before if not more active [22]. The third observation can be interpreted in the same way as earlier. Note that first-time infection occurs prior to any treatment. Hence, the estimate for the first-time infection is independent of the treatment effectiveness.

**Table 1:** **Estimated risk of per-encounter *C. trachomatis *** **acquisition (**$\bar{p}$**) (1st Model)**

**Age (yrs)**		**15**	**16**	**17**	**18**	**19**	**20**	**21**	**22**	**23**	**24**
$\bar{p}$	*q* = 0%	0.0585	0.0352	0.0243	0.0168	0.0110	0.0074	0.0052	0.0039	0.0024	0.0016
	*q* = 5%	0.0578	0.0344	0.0241	0.0167	0.0107	0.0072	0.0051	0.0034	0.0023	0.0015
	*q* = 10%	0.0569	0.0342	0.0239	0.0164	0.0106	0.0071	0.0050	0.0032	0.0022	0.0014

**Table 2:** **Estimated per-encounter acquisition risks of C. trachomatis causing first-time infection (**${\bar{p}}^{f}$**) and repeated infection (**${\bar{p}}^{r}$**) (2nd Model)**

**Age (yrs)**		**17**	**17.5**	**18**	**18.5**	**19**	**19.5**	**20**	**20.5**	**21**	**21.5**
${\bar{p}}^{f}$		0.0266	0.0216	0.0193	0.0169	0.0142	0.0122	0.0100	0.0075	0.0059	0.0049
${\bar{p}}^{r}$	*q* = 0%	0.0211	0.0133	0.0093	0.0070	0.0049	0.0038	0.0032	0.0028	0.0023	0.0018
	*q* = 5%	0.0185	0.0120	0.0086	0.0063	0.0045	0.0035	0.0029	0.0025	0.0021	0.0016
	*q* = 10%	0.0159	0.0102	0.0072	0.0055	0.0126	0.0031	0.0024	0.0021	00018	0.0044

We also computed the standard deviations of the estimates over multiple simulation runs for both models. All the standard deviations on the estimates over multiple simulation runs are small relative to the mean estimates. This implies that the cohort variation on the risk estimates is nearly negligible when using 10% as the maximum variation on the number of unprotected coital events.

We summarize the results in the following. First, our estimates are comparable to those in the literature. Based on the data collected from the same project, Tu et al. [38] estimated the per-encounter acquisition risk to be 0.009, which is between the estimates for 19 years old and 20 years old in our study. We think this is reasonable, considering the average enrollment age is 15 and average stay duration is 3.2 years. Kretzschmar et al. [15] estimated the upper bound for the per-contact probability of transmission to be 0.108 from male to female via casual sex contacts without condom use. Considering an approximately 7.6% of chlamydia prevalence in the age group of 15–39 cited by the authors in [15], we reasoned that the prevalence in the age group of 15 – 25 would be higher, approaching 10%. Then an upper bound on the acquisition risk based on their estimate would have been approximately 0.01. See Additional file 1 for a list of transmissibility estimates in the literature. Second, our results support the well-established evidence that age is an inverse independent predictor of chlamydial infection [51]. One of the most robust epidemiologic characteristics of chlamydial infection is higher prevalence among younger persons than older ones [50]. The inverse relationship between age and prevalence suggests that protective immunity is acquired over time. Third, our results support the hypothesis that protective immunity is partial at best [53]. Additional analysis with our data set also supported the evidence that repeated infections were strongly related with resumption of sexual activity [22], e.g., we found a similar level of sexual activity was resumed shortly after the treatment.

## Discussion

In this paper, we proposed a Markov-based individual state-transition model with age-varying transition probabilities to estimate age-specific risks of per-encounter *C. trachomatis* acquisition. To calibrate the model, we solved an optimization problem to identify the acquisition risks with which the simulated infection incidences match the observed ones at discrete age points. We conducted our case studies based on the data collected in a longitudinal study of recurrent STI among inter-city adolescent women. We further extended the model to study the differentiation between first-time infection and repeated infection.

There are a few issues that could potentially limit the use of the proposed method in practice. First, if an estimate on *C. trachomatis* transmissibility is requested, one must acquire knowledge on the prevalence of *C. trachomatis* among the male partner population for each age-dependent female population subgroup, which is essential to the conversion of age-specific acquisition risk into the age-specific transmission probability. Even in latest observational studies, it remains challenging to acquire accurate assessment on the *C. trachomatis* prevalence for the entire male partner population especially for the female cohort with extended casual sexual relationships. However, the proposed method provides a framework for “what-if” scenario analysis. Given hypothesized prevalence data, one can easily calculate the age-specific transmission probability and further conduct economic analysis on preventive programs/strategies. Second, our estimates are clearly affected by the accuracy of STI testing and behavioral reporting. It is worth noting that the researchers conducting the YWP undertook rigorous laboratory procedures to ensure the former accuracy. Meanwhile, Tu et al. [38] did not find much reporting bias among the study subjects when comparing self-reported coital counts attained from interviews at quarterly visits with those from subjects’ daily diaries in the same period. From a modeling point of view, inaccuracy of STI testing can be incorporated in the Markov state-transition model with updated transition probabilities for Pr (*s*_*i*+1_ = *C* | *s*_*i*_ = *C*) = ${\left(1-p_{i}\right)}^{n_{i}}+\left(1-{\left(1-p_{i}\right)}^{n_{i}}\right)\times q'$ where *q* ' is the false negative rate of the testing. As for modeling of behavior misreporting, since it is about the issue of mainly underreporting, a multiplier between 0 and 1 can be added to *n*(*t*) for the adjustment. Finally, we did not have data on the infection status of the sex partners of the study subjects. As a result, we were unable to build a finer-grained stochastic network type model that incorporates various aspects of inter-subject variations, e.g., we assumed a constant probability of having an infected partner for all sexual relationships. Ghani et al. [41] and Turner et al. [17] provide decent references on realistic sexual network models. Furthermore, it should be noted that we did assume a normal distribution on the number of sex acts among the study subjects. However, it was difficult to verify this assumption. Furthermore, we only varied the baseline for the subjects but did not incorporate potential differences on the temporal correlations of sex acts. A boarder class of distributions would be needed to protect the model from misspecification.

Despite of these limitations, we present a flexible and expandable platform for investigating various aspects of bacterial STI transmission. In our future research, we will address the aforementioned limitations with more systematic study of chlamydial infection data and more systematic analysis of realistic stochastic sexual network models.

## Competing interests

In the past five years, none of the authors have received reimbursements, fees, funding, or salary from an organization that may in some way gain or lose financially from the publication of this manuscript, either now or in the future. None of the authors hold any stocks or shares in an organization that may in some way gain or lose financially from the publication of this manuscript, either now or in the future. None of the authors is currently applying for any patents relating to the content of the manuscript. None of the authors has other financial competing interests.

## Authors’ contributions

YT carried out the mathematical model development, simulation input data analysis and model fitting. NK drafted the manuscript and made most of the revisions. WT provided the initial data for analysis and his expert opinion on model development and fitting. All authors read and approved the final manuscript.

## Supplementary Material

Additional file 1 ***C. trachomatis *** **transmissibility in the literature.** Click here for file

Additional file 2 **Parameter values of the cubic spline model **$n\left(t\right)=\exp\;\left\{\beta_{0}+\beta_{1}t+\beta_{2}t^{2}+\beta_{3}t^{3}+\sum_{i=1}^{N}u_{i}{\left({\left(t-k_{i}\right)}_{+}\right)}^{3}\right\}$**.** Click here for file
