# Supplementary material for: Estimating age-dependent per-encounter chlamydia trachomatis acquisition risk via a Markov-based state-transition model
Source: J Clin Bioinforma. 2014 Apr 25;4:7. doi: 10.1186/2043-9113-4-7 (PMC4022339; doi:10.1186/2043-9113-4-7)
Supplement: Additional file 1 — C. trachomatis transmissibility in the literature. [file 2043-9113-4-7-S1.doc]

**Additional file 1.** *C. trachomatis* transmissibility in the literature

| Value | Reference | Notes |
| --- | --- | --- |
| 0.108 | Kretzschmar M, Welte R, van den Hoek A, *et al.* 2001. Comparative model-based analysis of screening programs for Chlamydia trachomatis infections. *Am J Epidemiol* 153:90–101. | Upper bound for the per-contact probability of transmission.  Following the formula in Katz^1^, the authors did the estimation based on an estimated per partnership transmission probability in Quinn et al.^2^ and assumed average number of sex-act, *n* = 10.  *1. Katz BP. 1992. Estimating transmission probabilities for chlamydial infection. Stat Med* 11*:*565*–77.*  *2. Quinn TC, Gaydos C, Shepherd M, et al. 1996. Epidemiologic and microbiologic correlates of Chlamydia trachomatis infection in sexual partnerships. JAMA* 276*:*1737*–42.* |
| 0.09 | Ruijs GJ, Schut IK, Schirm J, *et al.* 1988. Prevalence, incidence, and risk of acquiring urogenital gonococcal or chlamydial infection in prostitutes working in brothels. *Genitourin Med* 64:49–51. | High-risk population |
| 0.14 | Lycke E, Lowhagen GB, Hallhagen G, *et al.* 1980. The risk of transmission of genital Chlamydia trachomatis infection is less than that of genital Neisseria gonorrhoeae infection. *Sex Transm Di*s 7:6–10. | Descriptive statistical analysis of observational data |
| 0.11 | Vickerman P, Watts C, Alary M, Mabey D, Peeling RW. 2003. Sensitivity requirements for the point of care diagnosis of Chlamydia trachomatis and Neisseria gonorrhoea in women. *Sex Transm Infect* 79:363-8. | Average of the above three estimates. |
| 0.0385 (steady, m-f);  0.0305 (steady, f-m);  0.154 (casual, m-f);  0.122 (casual, f-m) | Kretzschmar M, van Duynhoven YT & Severijnen AJ. 1996. Modeling prevention strategies for gonorrhea and Chlamydia using stochastic network simulations. *Am. J.* *Epidemiol* 144, 306–317. | Per-day transmission rate |
| 0.0375 | Turner KME, Adams EJ, Gay N, Ghani AC, Mercer C & Edmunds WJ. 2006. Developing a realistic sexual network model of chlamydia transmission in Britain. *Theor. Biol. Med. Model*. 3:3. | Range: 0.035-0.05  Developed a stochastic sex network and fitted it to the observed data |
| 0.154 (male to female);  0.122 (female to male) | Low N. et al. 2007. Epidemiological, social, diagnostic and economic evaluation of population screening for genital chlamydial infection. *Health Technol. Assess.* 11(8): iii–iv, ix–xii, 1–165. | Estimates taken from Kretzschmar et al. 2001 |
| 0.45 | Gray RT, Beagley KW, Timms P, and Wilson DP. 2009. Modeling the impact of potential vaccines on epidemics of sexually transmitted Chlamydia trachomatis infection. *Journal of Infectious Diseases* 199(11): 1680–1688. | Transmission probability per act at peak chlamydial load |
| 0.009 | Tu W, Ghosh P, Katz B. 2011. A stochastic model for assessing Chlamydia trachomatis transmission risk by using longitudinal observational data. *J. R. Statist. Soc. A.* 174(4): 975-989. | Per-encounter acquisition risk.  Without condom, if the prevalence rate among partners is 0.09, the estimated value of transmission probability is 0.1 with 95% CI (0.074,0.160); if the prevalence rate is 0.03, the estimate is 0.3 with 95% CI (0.223, 0.048). |
