# Supplementary material for: Estimating age-dependent per-encounter chlamydia trachomatis acquisition risk via a Markov-based state-transition model
Source: J Clin Bioinforma. 2014 Apr 25;4:7. doi: 10.1186/2043-9113-4-7 (PMC4022339; doi:10.1186/2043-9113-4-7)
Supplement: Additional file 2 — Parameter values of the cubic spline model nt=expβ0+β1t+β2t2+β3t3+∑i=1Nuit−ki+3. [file 2043-9113-4-7-S2.doc]

**Additional file 2**

Parameter values of the cubic spline model $n(t)=\exp\{\beta_{0}+\beta_{1}t+\beta_{2}t^{2}+\beta_{3}t^{3}+\sum_{i=1}^{N} u_{i}((t-k_{i})_{+})^{3}\}$

*β*_0_ = -41.26

*β*_1_ = 5.636

*β*_2_ = -0.245

*β*_3_ = 0.0036

| ***i*** | **1** | **2** | **3** | **4** | **5** | **6** | **7** | **8** | **9** | **10** |
| --- | --- | --- | --- | --- | --- | --- | --- | --- | --- | --- |
| ***k_i_*** | 14.75 | 15.18 | 15.45 | 15.73 | 15.94 | 16.15 | 16.33 | 16.53 | 16.71 | 16.88 |
| ***u_i_*** | 3.87e-15 | 1.54e-14 | 2.80e-14 | 4.56e-14 | 6.72e-14 | 9.76e-14 | 1.34e-13 | 1.89e-13 | 2.54e-13 | 3.29e-13 |
| ***i*** | **11** | **12** | **13** | **14** | **15** | **16** | **17** | **18** | **19** | **20** |
| ***k_i_*** | 17.10 | 17.27 | 17.47 | 17.66 | 17.84 | 18.01 | 18.19 | 18.38 | 18.57 | 18.73 |
| ***u_i_*** | 4.44e-13 | 5.57e-13 | 7.01e-13 | 8.53e-13 | 9.99e-13 | 1.15e-12 | 1.29e-12 | 1.43e-12 | 1.54e-12 | 1.61e-12 |
| ***i*** | **21** | **22** | **23** | **24** | **25** | **26** | **27** | **28** | **29** | **30** |
| ***k_i_*** | 18.92 | 19.11 | 19.30 | 19.52 | 19.74 | 19.94 | 20.16 | 20.39 | 20.61 | 20.82 |
| ***u_i_*** | 1.66e-12 | 1.68e-12 | 1.66e-12 | 1.60e-12 | 1.51e-12 | 1.39e-12 | 1.24e-12 | 1.06e-12 | 9.84e-13 | 7.32e-13 |
| ***i*** | **31** | **32** | **33** | **34** | **35** | **36** | **37** | **38** | **39** | **40** |
| ***k_i_*** | 21.03 | 21.26 | 21.52 | 21.82 | 22.10 | 22.41 | 22.78 | 23.13 | 23.51 | 24.05 |
| ***u_i_*** | 5.81e-13 | 4.25e-13 | 2.73e-13 | 1.35e-13 | 3.73e-14 | -3.2e-14 | -7.2e-14 | -7.7e-14 | -5.4e-14 | -1.3e-14 |
